# Genomic characterization and evolution of SARS-CoV-2 of a Canadian population

**DOI:** 10.1371/journal.pone.0247799

**Published:** 2021-03-04

**Authors:** Manna Zhang, Lin Li, Ma Luo, Binhua Liang

**Affiliations:** 1 Section of Hepatology, Department of Medicine, Health Sciences Centre, University of Manitoba, Winnipeg, MB, Canada; 2 JC Wilt Infectious Disease Research Centre, Public Health Agency of Canada, National Microbiology Laboratory, Winnipeg, MB, Canada; 3 Department of Medical Microbiology & Infectious Diseases, Rudy Faculty of Health Sciences, University of Manitoba, Winnipeg, MB, Canada; 4 Department of Biochemistry & Medical Genetics, Rudy Faculty of Health Sciences, University of Manitoba, Winnipeg, MB, Canada; Centers for Disease Control and Prevention, UNITED STATES

## Abstract

COVID-19 has greatly affected public health and world economy. In this study, we analyzed 129 full-length genomes of SARS-CoV-2 viruses of a Canadian population during early phase of the pandemic. Phylogenetic analysis revealed three major paths of transmission of SARS-CoV-2 viruses into Canada. Twenty-one substitutions that have frequencies greater than 3% of viral population were identified. Analysis of these substitutions indicated that P1427I (ORF1b), Y1464C (ORF1b), and Q57H (ORF3a) might affect functions of the corresponding SARS-CoV-2 encoded proteins. Additionally, we found the evidence of positive selection on the ORF3a and codon 614 of Spike protein, suggesting the viral components responsible for host entry and activation of inflammation response were targeted by host immune responses. The study showed genomic variation and evolution of SARS-CoV-2 in a Canadian population. These information may help develop preventive strategies and be used for further study of SARS-CoV-2 pathogenesis and therapeutics development.

## Introduction

The unknown pneumonia case was first reported on December 31^st^, 2019 in Wuhan, China, and a novel coronavirus, named 2019-nCoV (lately SARS-CoV-2), was later identified to be the etiological agent of the pneumonia [1, 2]. Within a month, this coronavirus disease (COVID-19) swept China and rapidly spread across the globe. As of May 18, 2020, close to five million cases and over 300,000 deaths have been reported in 210 countries/or territories around world (http://www.worldmeters.info/coronavirus/). The COVID-19 pandemic has seriously affected global health and world economy.

To date, no therapeutic interventions or vaccines have been proven effective against SARS-CoV-2 infection. Viral-host interactions will likely determine the outcome of SARS-CoV-2 infection. Thus, it is important to analy2ze viral mutations driven by host immune responses to understand how host immune response influence viral mutations.

To sustain infection in humans, SARS-CoV-2 viruses must have the ability to transmit from person-to-person efficiently. It requires the virus to adapt to the hosts and the interplay between viruses and host factors/or antiviral defence determines outcome of infection [3]. This interplay is linked to multiple biological processes of SARS-CoV-2 in hosts, mainly including its entry into hosts, replication, transcription, and translation where SARS-CoV-2 and its coding proteins interact with human proteins [4, 5]. As such, the novel coronaviruses are expected to be under a broad range of host immune pressures exerted by these host factors and mutate to evade host immune responses. For instance, Angiotensin-converting enzyme 2 (ACE2) as a cell receptor medicates the entry of the SARS-CoV-2 into host cells and is the critical determinant of viral host range, tropism, and infectivity [5, 6]. The selective pressures exerted by ACE2 on coronaviruses have been detected by several studies that a number of mutations in the receptor-binding domain (RBD) of SARS-CoV have been shown to contribute to the adaption of viruses to human cells [7–9]. Zinc finger antiviral protein (ZAP), apolipoprotein B mRNA editing-catalytic polypeptide-like 3 (APOBEC3), and adenosine deaminases acting on RNA (ADARs) protein are the well- known host antiviral proteins which are involved in viral replication. ZAP is able to suppress CpG dinucleotides, especially in the coding regions S and N proteins [10, 11] while APOBECV3/or ADARs constantly edits C to U in the transcriptome of SARS-CoV-2 [12]. As a result, host immune-driven mutations are imprinted in viral genomes during viral replication and evolution. It is believed that genomic variation is one of the important mechanisms of RNA virus evolution in hosts [13]. Investigation of genomic variation and evolutionary dynamics of novel coronavirus will help understand its origin, transmission, and pathogenesis.

In this study, 129 full-length genome sequences from a Canadian population were collected from the Global Initiative on Sharing Avian Influenza Database (GISAID). The genomic variation and evolution of SARS-CoV-2 as well as host immune pressure on viruses were investigated. This study aims to understand evolution of the SARS-CoV-2 virus and genetic mechanisms of pathogenesis which may help develop preventive and therapeutic interventions such as antivirals and vaccines.

## Materials and methods

### Viral sequences

The full-genome sequences with an average length of more than 29000 base pairs, high coverage, metadata, and annotations of SARS-CoV-2 were obtained from the Global Initiative on Sharing Avian Influenza Database—GISAID [14] and NCBI nucleotide sequence database—GenBank [15] on April 9^th^, 2020. Among them, 129 full-genome sequences are from Canada. The full names of the genome sequences are listed in **S1 Table.**

### Genomic analysis

Full-genome sequences were aligned using L-INS-I alignment method implemented in MAFFT v7.4.2 [16], setting data type as nucleic acids, gap extend penalty and opening penalty as default settings (0.123 and 1.53, respectively). The multiple alignments were manually edited and visualized using MEGA X v10.1 [17]. The open reading frames (ORFs) of the studied full-genome sequences were predicted and annotated by referring to complete genome generated from the earliest pneumonia virus isolate Wuhan-Hu-1(access number: MN908947.2) and were processed using Bio Edit v7.2 [18]. The genomic variants were identified referring to MN908947 using SNP-sites [19] and annotated using MEGA X v10.1. The frequencies of the identified variants were calculated using Perl Scripts developed in-house and plotted along genomic position of SARS-CoV-2 using Graph Pad Prism v8 (Graph Pad Software, Inc. San Diego California, USA). The similarity plots of the full-genome sequences from the different populations were performed based on the average pairwise scores calculated by moving a window of 4 nucleotides along the aligned sequences using Plotcon implemented in EMBOSS [20]. Functional effect of the identified amino acid substitutions on the corresponding proteins were predicted using Protein Variation Effect Analyzer (PROVEAN) on the assumption that protein sequences evolutionarily conserved among living organisms have survived natural selection [21]. The threshold score was set as a default value (-2.5) below which the mutation was predicted to have a “deleterious” effect.

### Phylogenetic reconstructions and analysis

Phylogenetic trees were built with Fast Tree [22] using approximate maximum-likelihood (ML) method with the aligned full-genome nucleotide sequences from the worldwide and Canadian populations. Before building phylogenetic trees, the best-fit nucleotide substitution model was determined using MEGA X v10.1. The generalised time reversible substitution model (GTR) was then selected as nucleotide evolution model based on the lowest Akaike Information Criterion (AIC) score it was generated. The tree topologies were validated according to scores of Shimodaira-Hasegawa (HS) tests with 1000 bootstraps implemented in FastTree. The scores over 0.7 indicate a high confidence in the given split of the tree/or subtree. All the generated phylogenetic trees were visualized in rooted (referring to MN908947) or unrooted view with Archaeopteryx v0.9928 beta [23].

### Inference of selective pressures

The nucleotide sequences of SARS-CoV-2 gene data sets, including (1). ORF1a; (2). ORF1b; (3). Spike (S); (4). ORF3a; (5). ORF8; (6). Nucleocapsid (N), were generated from the aligned full-genome nucleotide sequences using BioEdit. A combination of evolutionary analysis methods such as branch-site unrestricted statistical test for episodic diversification (BUSTED) and fast unconstrained Bayesian approximation (FUBAR) implemented in the Datamonkey and HyPhY [24] were then applied on the generated gene data sets to infer selection pressure on the various SARS-CoV-2 genes. BUSTED can test for positive selection on the specific gene that has experienced at least one site on at least one branch. A P value of likelihood-ratio test (LTR) less than 0.05 indicates evidence of gene-wide episodic diversifying (positive) selection. FUBAR employs a Bayesian approach to infer positive selection on individual sites for a given gene using posterior probabilities. Posterior probabilities over 0.9 are strongly suggestive of positive selection on the corresponding site of a given gene.

## Results

### Distribution of SARS-CoV-2 viruses

In this study, the 129 full-genome nucleotide sequences are primarily from Ontario (50.4%) and British Columbia (45.7%) of Canada collected on April 9^th^, 2020. Only about 4% of the total sequences are from other provinces, including Manitoba (1.6%), Saskatchewan (0.7%), New Brunswick (0.7%), and Nova Scotia (0.7%). There are no SARS-CoV-2 genome sequences from Alberta, Newfoundland and Labrador, Prince Edward Island, Quebec provinces, Yukon, Northwest, and Nunavut territories, although SARS-CoV-2 infection has been reported in these regions except Nunavut at the time. Thus, the full genome sequences included in the study are mostly from Ontario and British Columbia, which are affected early in the pandemic.

### Genomic variation of SARS-CoV-2 in Canada and its potential impact on functionalities of SARS-CoV-2

**Fig 1:**
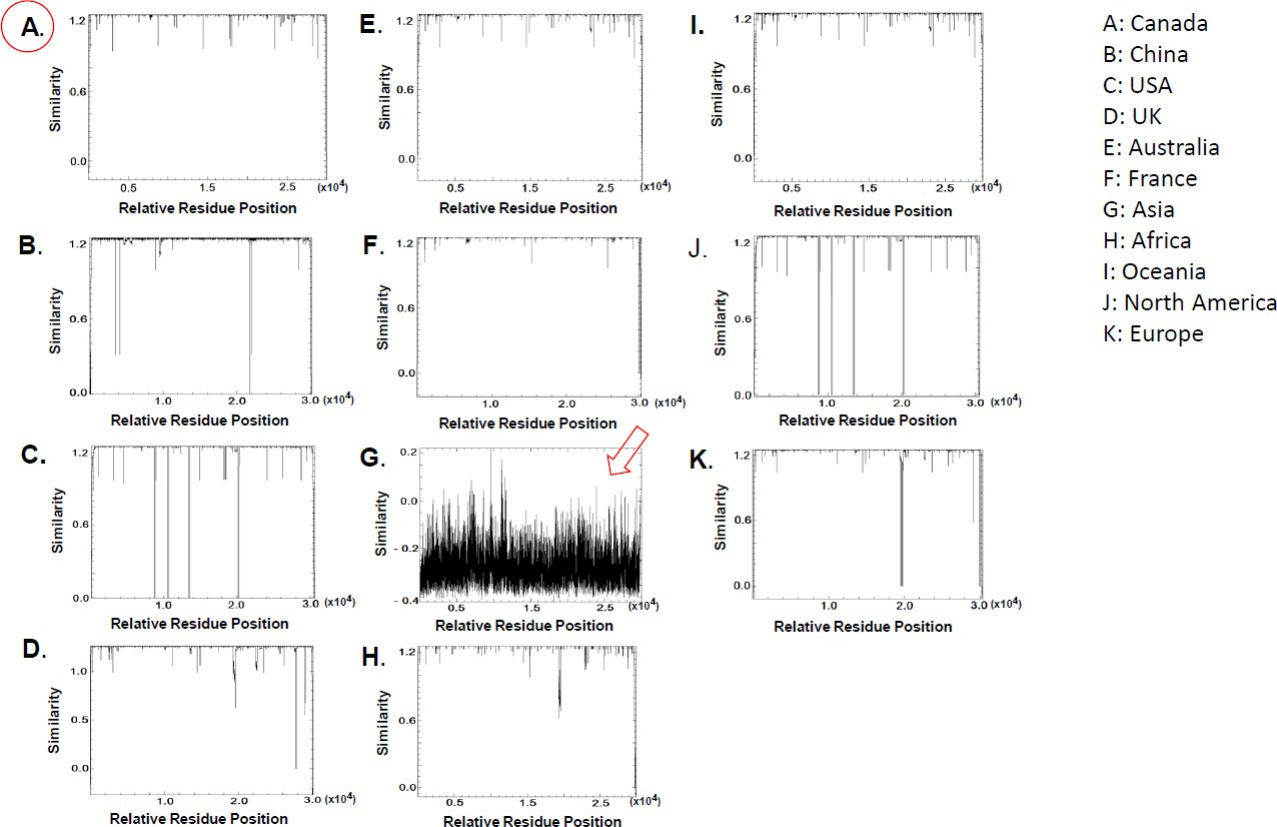
Sequence diversity of SARS-CoV-2 from different populations around world. Sequence similarity was plotted according to viruses from the corresponding populations using Pluton implemented in EMBOSS. Red circle shows the result from Canadian population; arrow shows the result from Asian population.

The single-stranded RNA genome size of SARS-CoV-2 viruses is in a range of 29,611–29,881 bps. To characterize the sequence diversity of SARS-CoV-2, we qualitatively measured the similarities of sets of aligned full-genome sequences from different populations. As shown in **Fig 1A**, there is a very high similarity (>0.8) amongst the aligned SARS-CoV-2 genomes from this Canadian population. Similar results were found in Australian (**Fig 1E**), France (**Fig 1F**), and Oceania (**Fig 1J**) populations. In contrast, lower similarities of SARS-CoV-2 genomes were observed in US (**Fig 1C**). The SARS-CoV-2 sequences in Asian (**Fig 1G**) populations have the lowest degree of similarity across the entire regions of SARS-CoV-2 genomes, indicating that SARS-CoV-2 sequences are very diverse in Asian populations.

**Fig 2:**
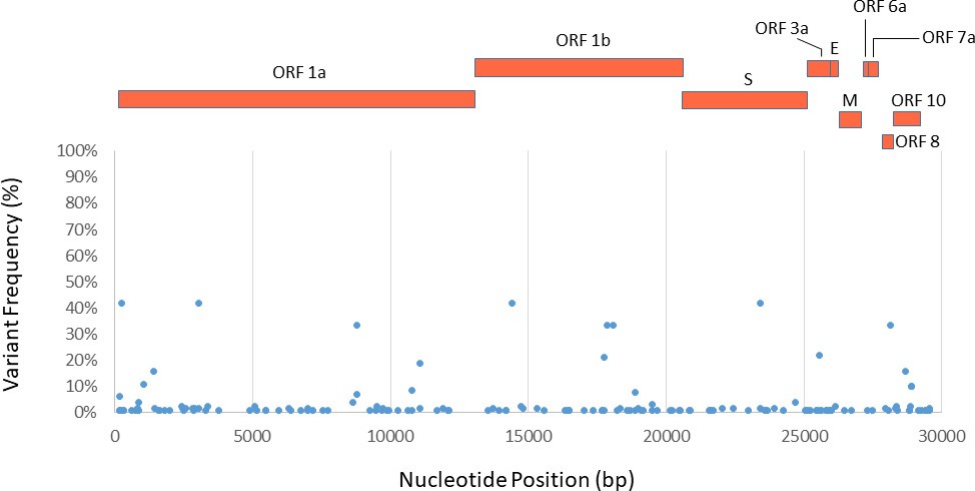
Genomic variation of SARS-CoV-2 in a Canadian population. Genomic variants were identified by referring to MN908947 using SNP-sites [10]. The locations and frequencies of the variants were plotted along genomic sequence of MN908947. The open reading frames of SARS-CoV-2 were shown as rectangles that were aligned with nucleotide positions of coronavirus.

To further characterize genomes of the novel coronaviruses in this Canadian population, we analyzed sequence variants across the entire genome of virus in comparing to MN908947. A total of 160 variants were identified across the entire SARS-CoV-2 genome (**S2 Table and Fig 1**). Among the identified variants, the majority (105 out of 160) of them are rare variants that have frequencies less than 1% of viral population. Only 21 variants (12%) have frequencies over 3% and are located in the coding regions as followings: (1). ORF1a (7); (2). ORF1b (6); (3). S (2); (4). ORF3a (1); (5). ORF8 (1); and (6). N (4) (**Table 1 and Fig 2**). Of them, the majority (12 out 21) of them are nonsynonymous mutations and only 9 are synonymous mutations, implying that may alter gene functions of the novel coronaviruses circulating in a Canadian population. Moreover, these variants mainly concentrate on ORF1ab and Nucleocapsid coding regions. No variants with frequencies over 3% were identified in E, M, ORF6, and ORF7a coding regions (**Fig 2**). Moreover, some variants such as ORF1a – 2772, ORF1b-941, and S-1841 have high frequencies (over 40%) in viral population (**Table 1**). These variants are also abundant in SARS-CoV-2 of many other countries, including US, UK, France, Belgium, Australian, New Zealand, and Iceland populations (**Table 2**). It is known that the spike protein plays a key role in host entry through binding to receptors on the host cell [5]. Mutations in spike, especially receptor biding domain (RBD), may change conformation and affect viral entry into host cell. Among all the nonsynonymous mutations, only S1841 (D614G) is located in Spike, but not in RBD region. This mutation has also been reported in a Germany population [25]. Three variants T8502C (ORFla), T10506C (ORF1a), and C6017T (ORF1b) found in this Canadian population were rare among other populations (frequencies < 1%) (**Table 2**).

**Table 1:** The identified variants of SARS-CoV-2.

CDS^a^	Position (Nt/Codon)	Reference^b^	Observed	Frequency (%)	AA^e^ change
1a	794 /265	C	T	10.85	T -> I
	1132 /378	G	A	15.5	V -> I
	**2772 /924**	**C**	**T**	**41.86**	**No**
	8502 /2834	T	C	6.98	No
	8517/2839	C	T	33.33	No
	10506/3502	T	C	8.53	No
	10818/3606	G	T	18.6	L -> F
1b	**941/314**	**C**	**T**	**41.86**	**P -> I**
	4280/1427	C	T	20.93	P -> L
	4391/1464	A	G	33.33	Y -> C
	4593/1531	C	T	33.33	No
	5410/1804	C	T	3.10	No
	6017/2006	C	T	7.75	No
S	**1841/614**	**A**	**G**	**41.86**	**D -> G**
	3133/1045	A	T	3.88	No
ORF 3a	171/57	G	T	21.71	Q -> H
ORF 8	251/84	T	C	33,33	L -> S
N	415/139	T	C	15.5	No
	608/203	G	A	10.08	R -> K
	609/203	G	A	10.08	R -> K
	610/204	G	C	10.08	G -> R

The genomic sequences of SARS-CoV-2 viruses from a Canadian population were aligned globally. The variants were identified by referring to MN908947. The variants with their frequencies over 3% were shown.

^a^ CDS–coding sequence region;

^b^ reference–MN908947;

^c^ Syn–synonymous mutation;

^d^ Non-Syn–nonsynonymous mutation;

^e^ AA–amino acid; ORF: open reading frame;

^f^ S–spike;

^g^ N–nucleocapsid. Variants with frequencies > 40% were bold.

**Table 2:** Comparison of the identified variants of SARS-CoV-2 between Canadian and other populations.

CDS^a^	Pos^b^ (Nt)	Ref^c^	Alt^d^	CA^e^ (%)	CN^f^ (%)	US^g^(%)	UK^h^ (%)	FR^i^ (%)	BE^j^(%)	AU^k^ (%)	IS^l^ (%)	NZ^m^ (%)
1a	794	C	T	10.8	0.79	32.9	2.89	29.3	8.71	14.7	20.1	5.08
	1132	G	A	15.5	4.74	0.22	0.54	0	0	8.47	0	0.85
	2772	C	T	41.8	0.40	45.8	61.0	92.6	89.4	46.2	78.8	80.5
	**8502**	**T**	**C**	**6.98**	**0.79**	**0**	**0**	**0**	**0**	**0**	**0**	**0**
	8517	C	T	33.3	35.2	44.8	0.54	1.06	0.38	22.1	4.09	4.24
	**10506**	**T**	**C**	**8.55**	**0**	**0.22**	**0**	**0**	**0**	**0.55**	**0**	**0**
	10818	G	T	18.6	7.11	0	21.7	4.26	3.03	28.1	13.8	14.4
1b	941	C	T	41.8	0.40	4.90	61.4	92.6	81.1	46.2	79.2	81.4
	4280	C	T	20.9	0	41.1	0.18	0	0	11.2	4.09	0
	4391	A	G	33.3	0	41.2	0	0	0	11.5	4.09	0
	4593	C	T	33.3	1.98	41.2	0	0	0	11.5	3.72	0
	5410	C	T	7.75	0	2.51	0.18	0	3.79	0.27	0	0
	**6017**	**C**	**T**	**3.10**	**0**	**0**	**0**	**0**	**0**	**0**	**0**	**0**
S^n^	1841	A	G	41.8	0.79	45.8	61.2	92.6	89.0	46.2	79.2	79.7
	3133	A	T	3.88	1.19	0	0	0	0	7.65	4.08	0
^o^ORF 3a	171	G	T	21.7	0.39	1.19	6.86	54.3	10.2	17.2	20.1	0.85
ORF 8	251	T	C	33.3	35.2	44.9	0.54	1.06	0	22.1	4.09	10.2
N^p^	415	T	C	15.5	4.35	0.22	0.36	0.38	0	8.19	0	0.85
	608	G	A	10.1	0	3.80	28.9	3.19	31.8	13.1	21.2	55.9
	609	G	A	10.1	0	3.70	28.9	3.19	31.8	12.8	21.2	55.9
	610	G	C	10.1	0	3.70	28.8	3.19	31.8	12.8	21.2	55.9

The variants identified from a Canadian population (n = 129) were compared to ones from other populations (sample sizes) as shown in terms of their frequencies in the corresponding populations. The variants with their frequencies over 3% were shown.

^a^ CDS–coding sequence region;

^b^ pos–position (nucleotide);

^c^ ref–reference (MN908947);

^d^ Alt–alterative;

^e^ CA–Canada (n = 129);

^f^ CN–China (n = 253);

^g^ US–United State (n = 918);

^h^ UK–United Kingdom (n = 554);

^i^ FR–France (n = 188);

^j^ BE–Belgium (264);

^k^ AU–Australia (n = 366);

^l^ IS–Iceland (n = 269);

^m^ NZ–New Zealand (n = 118);

^n^ S–Spike;

^o^ ORF—open reading frame;

^p^ N–nucleocapsid.

As many of the identified genomic variants are either first reported or their functional effects are unknown, we predicted functional effect of the identified nonsynonymous variants on SARS-CoV-2 using PROVEAN. We found that 3 out of 11 non-synonymous substitutions affected functions (deleterious) of SARS-CoV-2, including P1427L (ORF1b), Y1464C (ORF1b), and Q57H (ORF3a), implying potential changes of fitness of the strains harbouring these mutations (**Table 3**).

**Table 3:** Functional effect of the identified non-synonymous amino acids substitutions on the proteins of SARS-CoV-2.

CDS^a^	Position (AA^b^)	Reference^c^	Observed	Frequency (%)	PROVEAN Score^d^	Effect on Protein
1a	265	T	I	10.85	-0.841	Neutral
	378	V	I	15.5	-0.199	Neutral
	3606	L	F	18.6	-1.400	Neutral
1b	314	P	I	41.86	-0.797	Neutral
	**1427**	**P**	**L**	**20.93**	**-6.599**	**Deleterious**
	**1464**	**Y**	**C**	**33.33**	**-8.303**	**Deleterious**
S^e^	614	D	G	41.86	+0.598	Neutral
^f^ORF 3a	**57**	**Q**	**H**	**21.71**	**- 3.286**	**Deleterious**
ORF 8	84	L	S	33,33	+2.333	Neutral
N	203	R	K	10.08	-1.604	Neutral
	204	G	R	10.08	-1.656	Neutral

The non-synonymous amino acids substitutions were identified from a Canadian population by referring to MN908947. The substitutions with their frequencies over 3% were shown.

^a^ CDS–coding sequence region;

^b^ AA–amino acid;

^c^ reference–MN908947;

^d^ Substitutions with a score equal to or below– 2.5 are considered “deleterious”; otherwise considered “neutral”;

^e^ S–spike;

^f^ ORF–open reading frame;

^g^ N–nucleocapsid. The deleterious mutations were bold.

### Phylogenetic relationships among SARS-CoV-2 viruses

**Fig 3:**
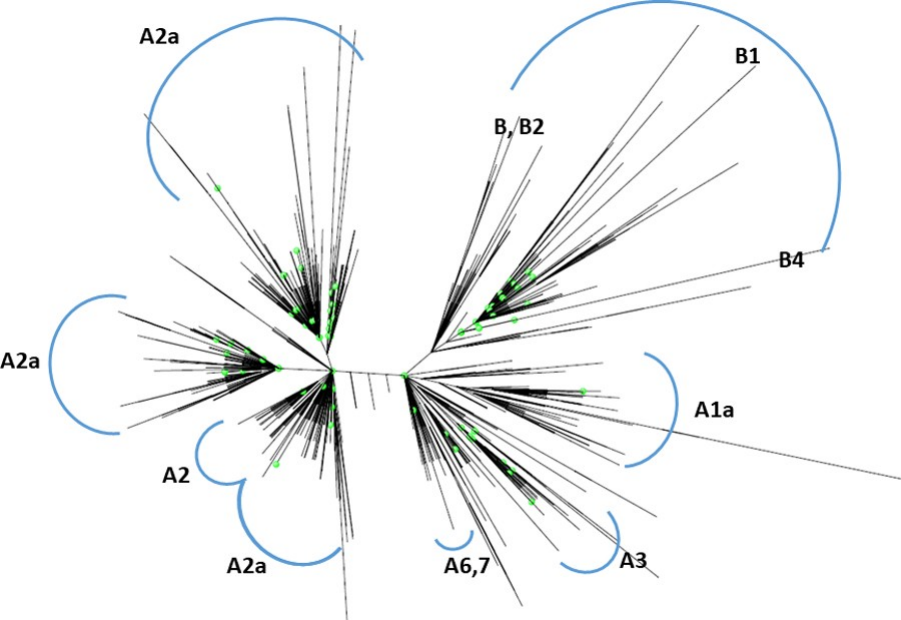
Phylogenetic relationship of the SARS-Cov-2 sequences of a Canadian population with other reported sequences in the world. Phylogenetic tree was built by an approximately maximum likelihood method on the full-genomes of novel coronaviruses worldwide from GISAID. The clades were defined by referring to the classification of Nextstrain (https://nextrain.org/). Canadian isolates were highlighted in green dots.

To infer origin of Canadian isolates, we built a whole approximating maximum likelihood phylogenetic tree using 3998 full-length genomes of SARS-CoV-2 viruses available on GISAID as of April 8^th^, 2020, including Canadian isolates and those across the world. In the tree, 129 viruses from a Canadian population were dispersed across 9 different SARS-CoV-2 lineages, including A1a, A2a, A3, A6, A7, B, B1, B2, and B4 (https://nextstrain.org/ncov/global?c=clade_membership) (**Fig 3**). This result showed that SARS-CoV-2 infection in Canada is caused by multiple viruses from different lineages.

**Fig 4:**
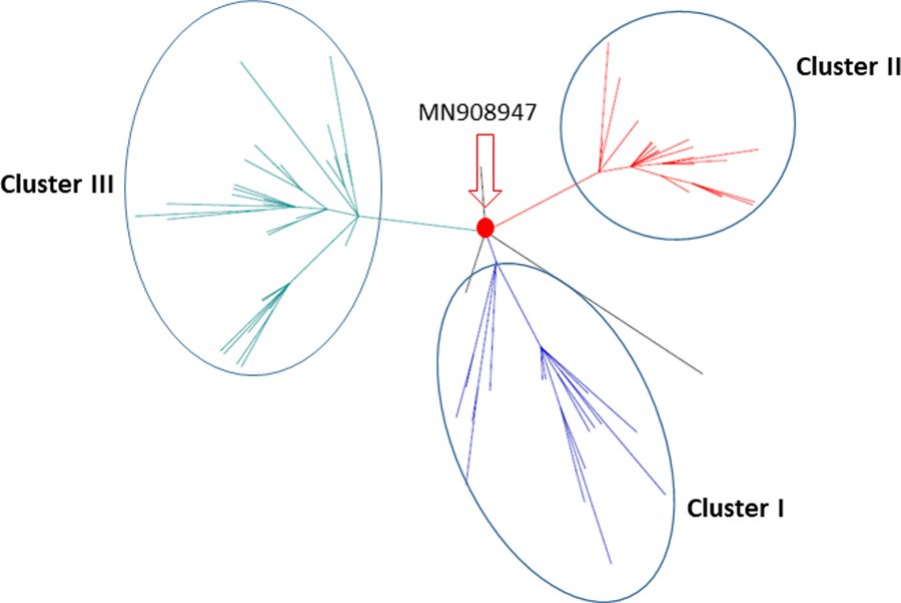
Phylogenetic tree of the SARS-Cov-2 sequences in Canada. Phylogenetic tree was built by an approximately maximum likelihood method on the full-genomes of novel coronaviruses from a Canadian population. The three groups of lineages were classified into three clusters: cluster I, II, and III. MN908947 is the reference. Red dot indicates the reference.

To evaluate potential divergence events and predict transmission paths of SARS-CoV-2, we reconstructed both rooted and unrooted evolution trees using only full-length genomes from Canadian isolates with the earliest virus from Wuhan, China (access No: MN908947) as reference. The results of the phylogenetic analysis demonstrated the rapid divergence of SARS-CoV-2 into three distinct transmission clusters (**Figs 4 and S1)**. Cluster I indicated the earliest divergent event as its common ancestor has the shortest branch length to MN908947 (S1 Fig). Cluster I was composed of almost equal numbers of viruses from ON and BC provinces. This result supports multiple introductions of SARS-CoV-2 into Canada. Cluster II was dominant by viruses from BC (62.3%). In comparison, Cluster III was composed of more diverse viruses across Canada, including ON, BC, MB, NB, SK, and NS although the majority of viruses (62.2%) were from ON. It is the largest cluster (n = 53) and represents the most recently divergent lineages in this Canadian population as the common ancestor of it has the longest branch length to MN908947 (S1 Fig).

### Host immune pressures on SARS-CoV-2 genes

As a RNA virus, the novel coronavirus has a relatively high mutation rate and each virus is a cloud of related mutants–quasispecies which are restricted by host selection (host immune responses) pressure. To identify and quantify positively selected mutations, we applied a branch-site unrestricted statistical test for episodic diversification–BUSTED method. This approach is able to detect positive selection that accumulates across multiple sites of the tested gene with a higher statistical power than a single site of the tested gene where the selection may not be detected [26]. The results showed that only ORF 3a gene was under positive selection (*P* = 0.001), and no evidence of positive selection (*P* > 0.05) was found on other genes of SARS-CoV-2 (**Table 4**). Two sites: OFR3a – 145 and 186 trend towards positive selection (evidence ratio: 51.49/5.09 and 30.71/3.34), but the result is not statistically significant.

**Table 4:** Tests results for a gene-wide or a per-site based positive selection on SARS-CoV-2.

CDS^a^	BUSTED^b^				FUBAR^c^		
	Gene-wide PS^d^	P value^e^	Position (AA)	ER^f^ (Con/Uncon)	Position (AA)	Posterior probability (β>α)	Bayes Factor
1a	No	0.5	NA	NA	NA	NA	NA
1b	No	0.5	NA	NA	NA	NA	NA
**S**	No	0.5	NA	NA	**614**	**0.938**	**18.26**
**ORF**^g^ **3a**	**Yes**	**0.001**	145	51.49/5.09	**186**	**0.937**	**17.56**
			186	30.71/3.34			
ORF 8	No	0.166	NA	NA	NA	NA	NA
N^f^	No	0.5	NA	NA	NA	NA	NA

Tests were conducted to infer selection pressure on SARS-CoV-2 using BUSTED and FUBAR methods implemented in HyPhy.

^a^ coding sequence region;

^b^ BUSTED–branch-site unrestricted statistical test for episodic diversification;

^c^ FUBAR–fast unconstrained Bayesian approximation;

^d^ EDS–positive selection;

^e^ likelihood-ratio test;

^f^ ER–evidence ration (constrained model/un-constrained model);

^g^ ORF–open reading frame;

^f^ N–nucleocapsid. Positively selected genes or sites are bold.

To identify positive selection on individual sites among genes of SARS-CoV-2, we used a Bayesian approach–FUBAR using posterior probabilities but not p-values [27]. This method is suitable for positive selection detection when it exists but relatively weak in p value. Evidence of positive selection was detected at codon 614 of the spike gene and at codon 186 of the ORF3a gene. Interestingly, amino acid change at codon 614 of the spike gene is at a very high frequency (41.86%) among the viruses in this Canadian population, implying that this mutation might help the spread of viruses among Canadian population. In addition, Positive selection at codon 186 of the ORF3a was suspected using BUSTED method and was confirmed by FUBAR. However, the frequency of the mutation at this codon was low (< 1%) in this Canadian SARS-CoV-2 viral population and the result requires validation.

## Discussion

In this study, we characterized genomic variation, phylogenetic and evolutionary dynamics of SARA-CoV-2 from a Canadian population during the first three months of the COVID-19 pandemic. The phylogenetic analysis showed multiple and unrelated SARS-CoV-2 lineages circulating in Canada. Similar results were also found in US [28], Italian [29], and Icelandic [30] populations. The novel coronaviruses from this Canadian population were classified to all currently defined 9 clades across world. It suggests that the spread of viruses into Canada via different routes. In Canada, around 26% of COVID-19 cases have been related to travel exposure and the first case of COVID-19 was also attributed to travel to Wuhan, China before returning to Canada [31]. It was estimated that the majority of the travellers to Canada were from Europe (42%) and Asia (35%), where COVID-19 outbreak occurred at an earlier time [31]. Thus, the results of our phylogenetic analysis are consistent with the report that the earlier COVID-19 cases are attributed to travel-related exposure while later new cases are increasingly being attributed to community transmissions after international travel to Canada is restricted for all foreign nationals from all countries. However, we cannot exclude undetected (cryptic) transmissions of multiple founder viruses in Canada because of limited sample size and most of the SARS-CoV-2 sequences are from BC and ON. Despite these limitations, our result demonstrated the importance of the early travel restrictions, especially from China and Europe to Canada, to prevent COVID-19 pandemic.

SARS-CoV-2 viruses from this population in Canada were classified into three distinct transmission clusters. It indicates three potential divergence events of SARS-CoV-2 in the past, and suggests possible transmission paths of early SARS-CoV-2 in Canada. The earliest divergent viruses formed cluster I which were solely composed of isolates from ON and BC provinces. ON and BC are the entry points for international travellers from Europe and Asia. Cluster III was the largest cluster (n = 53) and represented the most recent lineages in Canadian population in which more diverse viruses were found from all the provinces of Canada. Interestingly, there are a number of lineages such as BC_40860/2020, BC_35720, ON_PHL7512/2020, ON_PHL4181, ON-VIDO-01, and ON-PHL2445 which cannot be classified into any cluster above. These lineages were not related with each other or with others either, suggesting that they might not be transmitted within Canada. We checked genomic variation of these viruses and didn’t find deleterious mutations which affected functionalities of SARS-CoV-2 proteins. This result implies that these viruses were either transmitted to “dead-end” hosts or transmissions were broken by hosts who took preventive interventions. Most importantly, the lineages in Cluster III account for almost half of viruses isolates in Canadian population and represents not only the most recent lineages but also ones more abundant across Canada. These viruses may be more relevant for developing therapeutics and preventions.

The genome sequence diversity in the viruses from this Canadian population is very low compared to ones from other viral populations of the Countries that are more severely affected by the SARS-CoV-2 pandemic at the time, including US, China, and UK. The less diverse genomes of SARS-CoV-2 in this Canadian population are likely attributed to both a lower mutation rate of coronaviruses than all other RNA viruses because of their first known proofreading activities due to a 3’-to-5’ exoribonuclease [32] and relatively simple lineages circulating in the Canadian population at the time. In comparison, the highest sequence diversity of coronaviruses was found in Asia population. It suggests a more complex and diverse lineages in that area, implying the possible source of SARS-CoV-2.

SARS-CoV-2 virus was evolving during the early phase of COVID-19 pandemic in Canada. We observed 21 genomic mutations with frequencies over 3% of viral population. Over 50% of them changed coding sequences but only 3 mutations—P1427I (ORF1b), Y1464C (ORF1b), and Q57H (ORF3a), potentially affected the fitness of viruses. Under host immune pressure these deleterious mutations may change the phenotype of viruses and are eliminated by the positive (purifying) selection [33]. However, we didn’t detect positive selection on these sites using FUBAR possibly due to limited sample size or very weak selection effect that is hard to measure [26]. This was supported by our positive selection analysis on the whole ORF3a gene which was positively selected but none of the high frequency mutation sites was positively selected. It is possible that this is a transient or localized selective event and is difficult to measure (low statistical power) [34]. These mutations are also abundant in US, UK, French, Australian, Belgium, and Icelandic populations. Among them, P1427I (ORF1b) and Y1464C (ORF1b) have been reported to be signature mutations in USA in comparison to ones in Asian and European continent [35]. These sites may be potential targets for developing therapeutic agents and deserve further functional studies. In addition, replacement of Leucine with Serine at 84 position of ORF8 was found to have significant effect on the structure of the protein C-terminal which was possibly a phosphorylation target for the human Serine/Threonine kinases [36]. Interestingly, three substitutions: T8502C (ORF1a), T10506C (ORF1a), and C6071T (ORF1b) are only presented in viruses from this Canadian population but not in other populations in the world. Thus, these substitutions may be the signature mutations of viruses of this Canadian population. All of them are synonymous mutations.

Like other human CoVs (hCoVs), SARS-CoV-2 viruses are under host immune pressure and thus mutate to evade immune responses. These host immune-driven changes (mutations), especially positive (diversifying) selection, are potential good targets for therapeutic intervention as these mutations may affect fitness of viruses. In our analysis, the whole ORF3a protein was found to be positively selected. SARS-CoV ORF3a has been associated with the exceedingly high expression of cytokines and chemokines which is linked to a “pro-inflammatory cytokine storm” causing cell death that drives in part the high pathogenicity of SARS-CoV and MERS-CoV [3]. SARS-CoV ORF3a not only promoted NLRPs inflammasome assembly through TRAF3-dependent K63 ubiquitnation of ASC [37] but also provided a potassium flux through its ion channel domain to activate NEK7-dependent NLRP3 inflammasome [38]. Like other hCoVs, it is suspected that SARS-CoV-2 ORF3a serves the similar functions as SARS-CoV’s. In this regards, ORF3a may be an ideal target for developing therapeutic agents to modulate the activation of pro-inflammation and cell death programmes selectively. Another identified positive selection is at 614 (AA) of Spike protein. It has been reported in the previous study [25]. Although this site is not in the receptor binding domain (RBD), D614 is located on the surface of S protein promoter that contacts with the neighboring promoter through a Asp-85-Thr-89 hydrogen bond [39]. The binding may bring together the promoters of S1 and S2 units while D614G mutation possibly interrupts the interaction between the S1, and S2 units, blocking shedding of S1 from S2. In addition, D164 is embedded in a linear epitope of SARS-CoV S spike that mediates antibody-dependent enhancement (ADE) of coronavirus infection [40]. The ADE target peptide is identical to the region of SARS-CoV S_611-617_ that is neighboring the RBD. The antibody binding possibly changes the conformation of Spike protein that increases RBD-ACE2 interaction, resulting in an enhanced transmission of SARS-CoV-2 into hosts. More experimental studies are needed to investigate its functional effect on Spike that may affect virus entry into host. If it does, this site may also be a good target for therapeutic agents as viruses evolve towards harboring this substitution in host.

## Conclusion

We have characterized the genomic variation and evolutionary dynamics of SARS-CoV-2 from a Canadian population during the early phase of pandemic. Our findings have shed light on its early transmission in Canada. More importantly, we have identified unique genomic features (or patterns) of the virus may associated with pathogenicity of COVID-19 which may be potential targets for therapeutic interventions.

## Supporting information

S1 Fig Phylogenetic tree of the SARS-Cov-2 sequences in Canada. Phylogenetic tree was built by an approximately maximum likelihood method on the full-genomes of novel coronaviruses from a Canadian population. The tree was rooted by MN908947. The three groups of lineages were classified into three clusters: cluster I, II, and III.
(PDF)

S1 Table The names and access IDs of viruses from a Canadian population used in this study. (XLSX)

S2 Table The identified variants and their frequencies. (XLSX)
