# Supplementary material for: Genomic characterization and evolution of SARS-CoV-2 of a Canadian population
Source: PLoS One. 2021 Mar 4;16(3):e0247799. doi: 10.1371/journal.pone.0247799 (PMC7932534; doi:10.1371/journal.pone.0247799)

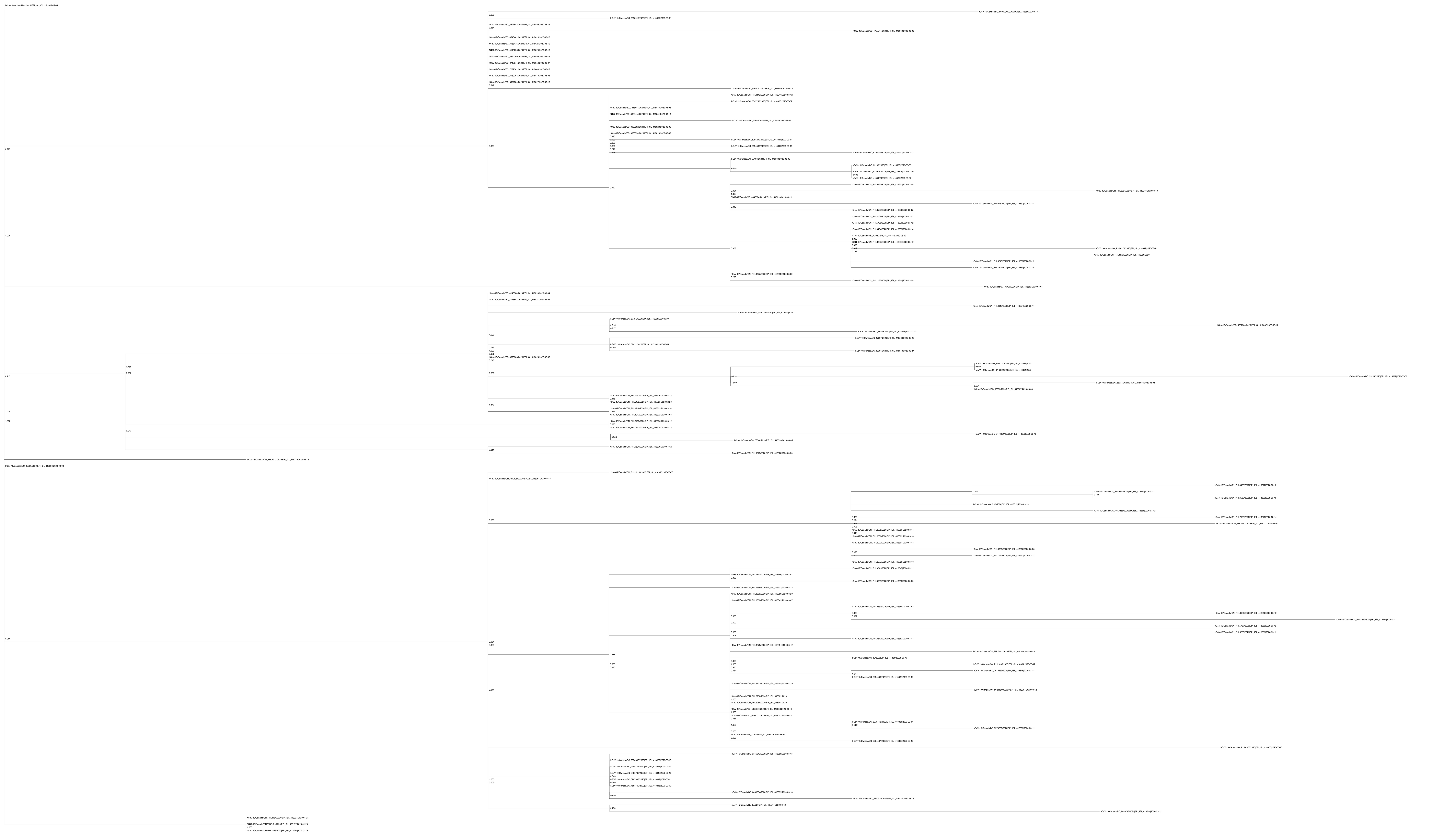

Supplement: S1 Fig — Phylogenetic tree was built by an approximately maximum likelihood method on the full-genomes of novel coronaviruses from a Canadian population. The tree was rooted by MN908947. The three groups of lineages were classified into three clusters: cluster I, II, and III. (PDF) [file pone.0247799.s001.pdf]
